# Discovery and Evolution of New Domains in Yeast Heterochromatin Factor Sir4 and Its Partner Esc1

**DOI:** 10.1093/gbe/evz010

**Published:** 2019-01-22

**Authors:** Guilhem Faure, Kévin Jézéquel, Florian Roisné-Hamelin, Tristan Bitard-Feildel, Alexis Lamiable, Stéphane Marcand, Isabelle Callebaut

**Affiliations:** 1 Sorbonne Université, Muséum National d’Histoire Naturelle, UMR CNRS 7590, IRD, Institut de Minéralogie, de Physique des Matériaux et de Cosmochimie, IMPMC, Paris, France; 2 Institut de Biologie François Jacob, IRCM/SIGRR/LTR, INSERM U1274, Université Paris-Saclay, CEA Paris-Saclay, Paris, France; 3 National Center for Biotechnology Information, National Library of Medicine, National Institutes of Health, Bethesda, MD; 4 Sorbonne Université, UMR CNRS 7238, IBPS, Laboratoire de Biologie Computationnelle et Quantitative (LCQB), Paris, France

**Keywords:** H-BRCT, Sir4, Itc1, Esc1, Asf2, Dbf4, mating-type switching, hydrophobic cluster analysis

## Abstract

Sir4 is a core component of heterochromatin found in yeasts of the *Saccharomycetaceae* family, whose general hallmark is to harbor a three-loci mating-type system with two silent loci. However, a large part of the Sir4 amino acid sequences has remained unexplored, belonging to the dark proteome. Here, we analyzed the phylogenetic profile of yet undescribed foldable regions present in Sir4 as well as in Esc1, an Sir4-interacting perinuclear anchoring protein. Within Sir4, we identified a new conserved motif (TOC) adjacent to the N-terminal KU-binding motif. We also found that the Esc1-interacting region of Sir4 is a Dbf4-related H-BRCT domain, only present in species possessing the HO endonuclease and in *Kluveryomyces lactis*. In addition, we found new motifs within Esc1 including a motif (Esc1-F) that is unique to species where Sir4 possesses an H-BRCT domain. Mutagenesis of conserved amino acids of the Sir4 H-BRCT domain, known to play a critical role in the Dbf4 function, shows that the function of this domain is separable from the essential role of Sir4 in transcriptional silencing and the protection from HO-induced cutting in *Saccharomyces cerevisiae*. In the more distant methylotrophic clade of yeasts, which often harbor a two-loci mating-type system with one silent locus, we also found a yet undescribed H-BRCT domain in a distinct protein, the ISWI2 chromatin-remodeling factor subunit Itc1. This study provides new insights on yeast heterochromatin evolution and emphasizes the interest of using sensitive methods of sequence analysis for identifying hitherto ignored functional regions within the dark proteome.

## Introduction

Heterochromatin is a feature of eukaryotic chromosomes with conserved roles in gene expression, genome spatial organization, and chromosome stability. In most eukaryotes, heterochromatic domains are decorated by histones H3 methylated on lysine 9 and bound by the heterochromatin protein HP1 (Larson et al. 2017; Strom et al. 2017; Allshire and Madhani 2018; Machida et al. 2018). These domains are usually large and can comprise up to half of the genome. Among eukaryotes, some yeast species do not share these two hallmarks. In the most studied budding yeast *Saccharomyces cerevisiae*, heterochromatin (also called silent chromatin) is restricted to a small fraction of the genome (<1%). It lacks the histone H3 lysine 9 methylation and HP1. Instead, its core components are H4 lysine 16 deacetylated nucleosomes bridged by the histone-binding factor Sir3 in stoichiometric complex with the protein Sir4 and the histone deacetylase Sir2 (Behrouzi et al. 2016; Gartenberg and Smith 2016). Despite these differences, other general properties of heterochromatin are conserved such as *cis* and *trans* cooperativity in the establishment of a repressive compartment for transcription, clustering at the nuclear periphery and near the nucleolus, epigenetic variegation, and late replication (Meister and Taddei 2013; Tatarakis et al. 2017).

The main function of heterochromatin in *S.cerevisiae* relates to mating, the ability of two haploid cells of opposite mating type (a and α) to form a diploid zygote. The *MAT* locus specifies the haploid cell mating type. To favor mating, a programmed DNA rearrangement allows a reversible switch from one mating type to the other (Hicks and Herskowitz 1976; Strathern et al. 1982; Haber 2012; Hanson and Wolfe 2017). In this process, DNA at the *MAT* locus is cleaved by the endonuclease HO, removed and replaced with DNA from either the *HML* or *HMR* locus. *HML* and *HMR* are ∼3-kb-long silent cassettes that store the sequence information specific of each mating type. Sir2/Sir3/Sir4-dependent heterochromatin prevents *HML* and *HMR* transcription and protects them from cleavage by HO during mating-type switching.

Heterochromatin in *S.cerevisiae* also covers a few kilobases at each telomere (Gartenberg and Smith 2016; Hocher et al. 2018). This subtelomeric heterochromatin has several functions. It clusters telomeres at the nuclear periphery and generates a local environment favoring heterochromatin at *HML* and *HMR* because both loci are not far from a telomere (∼15 and ∼25 kb, respectively). It also acts as a molecular sink ensuring that Sir proteins do not bind promiscuously at other sites in the genome (Buck and Shore 1995; Maillet et al. 1996; Marcand et al. 1996; Ruault et al. 2011; Meister and Taddei 2013; Guidi et al. 2015). Finally, subtelomeric heterochromatin contributes to genome stability. It prevents loss-of-heterozygosity by break-induced recombination at subtelomeres (Batté et al. 2017). It also inhibits telomere fusions by nonhomologous end joining (NHEJ) (Marcand et al. 2008) and favors telomere elongation through telomerase recruitment (Chen et al. 2018), two functions relying more specifically on Sir4, not Sir3 and Sir2.

As Sir4 binds each of the other Sir proteins, it is generally considered as a scaffold for the Sir complex assembly. Two structural domains have been recognized to date in Sir4, which are directly implicated in the Sir complex assembly. First, the extreme C-terminal end (amino acid [aa] 1271–1346) folds into a helical coiled-coil, allowing the protein to dimerize (Chang et al. 2003; Murphy et al. 2003) and to interact with Sir3 (Moretti et al. 1994; Moazed et al. 1997; Park et al. 1998). Second, a central domain, called the Sir2 interaction domain (SID, aa 737–893), makes contact with Sir2 (Moazed et al. 1997; Cockell et al. 2000; Ghidelli et al. 2001; Hoppe et al. 2002; Hsu et al. 2013), stimulating its deacetylase activity (Tanny et al. 2004; Cubizolles et al. 2006; Hsu et al. 2013).

In addition, both the N- and C-terminal regions of Sir4 target the Sir complex to chromatin, through association with other proteins, among which the DNA-binding factor Rap1 (Moretti et al. 1994; Luo et al. 2002) and the two subunits of the KU end-binding factor (Tsukamoto et al. 1997; Laroche et al. 1998; Martin et al. 1999; Mishra and Shore 1999; Luo et al. 2002; Roy et al. 2004; Taddei et al. 2004; Ribes-Zamora et al. 2007; Hass and Zappulla 2015; Chen et al. 2018). KU binding to Sir4 contributes to telomerase recruitment and the subsequent lengthening of telomeres (Chen et al. 2018; Hass and Zappulla 2015). Sir4 also participates in the anchoring of yeast telomeres at the nuclear periphery through its “partitioning and anchoring domain” (PAD, aa 960–1150), which interacts with the peripheral membrane anchor Esc1 (Ansari and Gartenberg 1997; Andrulis et al. 2002; Taddei et al. 2004). The same Sir4 PAD additionally interacts with the integrase of the Ty5 yeast retrotransposon, an interaction essential for the preferential integration of Ty5 in heterochromatin (Zhu et al. 2003; Kvaratskhelia et al. 2014). No structural information is, however, available for the Sir4 PAD and Esc1.

**Fig. 1.**
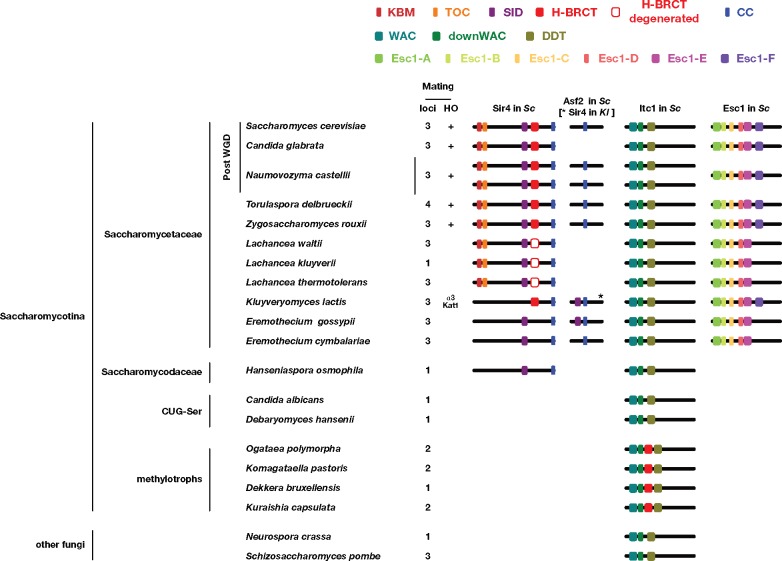
—Schematic view of the structural and functional domains identified in Sir4, Asf2, Itc1, and Esc1 in yeast species. Number of mating loci from Gordon et al. (2011) and Wolfe and Butler (2017). The empty red square represents the degenerated H-BRCT domains present in species from the *Lachancea* clade.

On an evolutionary point of view, Sir4 is the least conserved protein among the Sir proteins. It seemed to coappear in evolution with the two silent mating-type cassettes, that is, the three *MAT*-like-loci system found in the *Saccharomycetaceae* family of yeasts (including *Candida glabrata, Naumovozyma castellii, Zygosaccharomyces rouxii, Kluyveryomyces lactis*, and *Lachancea waltii*) (Génolevures Consortium 2009; Dujon 2010; Gordon et al. 2011; Wolfe and Butler 2017) (fig. 1). Within the larger subphylum *Saccharomycotina*, yeast species from the CUG-Ser clade (including *Candida albicans* and *Debaryomyces hansenii*) have only one *MAT* locus, no silent cassette, and apparently lack Sir4 (Bennett and Johnson 2005). The more distant yeasts of the methylotrophs clade often display a two *MAT*-like-loci system with only one silent cassette adjacent to a telomere or a centromere but also lack Sir4 (De Schutter et al. 2009; Kuberl et al. 2011; Curtin et al. 2012; Piskur et al. 2012; Morales et al. 2013; Ravin et al. 2013; Hanson et al. 2014, 2017; Maekawa and Kaneko 2014; Coughlan et al. 2016; Hanson and Wolfe 2017). How silencing is achieved in these yeasts is unknown.

A large part of the Sir4 protein sequence, including regions which are known to interact with partners, remains in the dark, that is, lacks annotations relative to known domain databases. In fact, such orphan sequences represent a significant portion of the whole proteomes (Faure and Callebaut 2013a; Bitard-Feildel and Callebaut 2017). A large part of this dark proteome corresponds to foldable regions, which once delineated, can be in some cases linked to already known families of domains, or are at the basis of the definition of novel families of domains (Faure and Callebaut 2013a; Bitard-Feildel and Callebaut 2017). These “hidden” relationships can be identified especially using fold signatures, which are more conserved than the sequences (Faure and Callebaut 2013b).

Using computational methods we previously developed (Faure and Callebaut 2013a, 2013b), we identified new domains or motifs in Sir4 and its partner Esc1. Particularly, we identified significant similarities of the Sir4 PAD with the conserved H-BRCT domain of Dbf4, the regulatory subunit of the Dbf4-dependent kinase (DDK or Cdc7) that is essential to replication initiation in eukaryotes. Dbf4 H-BRCT interacts in *S. cerevisiae* with an FHA domain of the Rad53^CHK2^ checkpoint kinase and constitutes a particular subfamily of BRCT (BRCA1 C-terminus) domains (Gabrielse et al. 2006; Matthews et al. 2012, 2014; Chen et al. 2013). The H-BRCT domain, hitherto uniquely found in Dbf4, has additional elements at the N- or C-terminus of a conserved BRCT core (Koonin et al. 1996; Callebaut and Mornon 1997; Leung and Glover 2011), which are critical to its specific function. We also identified H-BRCT domains within Itc1, a component of the conserved chromatin remodeling complex ISWI2 in Sir4-free species belonging to the methylotrophs clade. This study thus opens new perspectives on yeast heterochromatin evolution and for characterizing the role of H-BRCT domains, whose presence goes beyond the single Dbf4 protein.

## Materials and Methods

### Sequence Analysis

Hydrophobic cluster analysis (HCA) is well adapted to predict foldable domains (i.e., regions with a high density in hydrophobic clusters, mainly corresponding to the regular secondary structures) (Faure and Callebaut 2013a; Bitard-Feildel and Callebaut 2017) and to highlight within these foldable domains’ structural invariants, which are much more conserved than amino acid sequences (Faure and Callebaut 2013b; Bitard-Feildel et al. 2015). It is thus useful for deciphering information within “orphan” sequences, including well-folded domains for which it is possible to identify remote relationships (as for the H-BRCT domain highlighted here), as well as intrinsically disordered regions undergoing disorder-to-order transitions (as for the KU-binding motif [KBM] and TOC motif also described here) (Bitard-Feildel et al. 2018). Additional disorder predictions were made using IUPred (Dosztányi et al. 2005a, 2005b), completed by predictions of disorder-to-order transitions through the ANCHOR program (Dosztányi et al. 2009; Mészáros et al. 2009).

Sequence similarity searches were performed using PSI-BLAST (Altschul et al. 1997) and HHblits (Remmert et al. 2011), combined with HCA (Gaboriaud et al. 1987; Callebaut et al. 1997). Searches for sequence similarities with known 3D structures were made using HHpred (Meier and Söding 2015) and Phyre (Bennett-Lovsey et al. 2008).

The Yeast Gene Order Browser (http://ygob.ucd.ie/; last accessed February 2018) (Byrne and Wolfe 2005) was also used to visualize the syntenic context of the different genes.

Manipulation of 3D structures were performed using Chimera (Pettersen et al. 2004), whereas sequence alignment rendering was made using ESPript (Gouet et al. 1999).

## Results

### Analysis of the Whole Sir4 Sequence

Using HCA and SEG-HCA (Faure and Callebaut 2013a) (see Materials and Methods for the principles of the method) and starting from the only information of the single amino acid sequence of *S. cerevisiae* Sir4, we identified several regions predicted to consist of foldable domains (supplementary fig. S1, Supplementary Material online). Up to aa 720, hydrophobic clusters are scarce within globally charged regions, suggesting that this amino-terminal half of *S. cerevisiae* Sir4 should be globally disordered. Such a prediction is supported by disorder predictors, such as IUPred (Dosztányi et al. 2005a, 2005b) (supplementary fig. S1, Supplementary Material online). However, several regions in this amino-terminal half part have a higher density in hydrophobic clusters (supplementary fig. S1, Supplementary Material online), the lengths of which match those of regular secondary structures. Although these regions should not form on their own stable 3D structures, they may adopt such structures after energetic stabilization (typically upon binding with a partner) (Faure and Callebaut 2013a; Bitard-Feildel et al. 2018). This hypothesis is supported by ANCHOR results, which predicted disorder-to-order transitions in the same regions (Dosztányi et al. 2009; Mészáros et al. 2009) (supplementary fig. S1, Supplementary Material online). From aa 720 onward, the Sir4 sequence contains two regions that are predicted as globular, which can be further subdivided using SEG-HCA into seven foldable domains. The two first ones (aa 737–910) correspond to the SID, with the hinge separating the two regions (aa 786–802) matching the disordered segment of the domain (see below). The following ones (third to seventh) extend from aa 970 onward. The two last regions correspond to the coiled-coil domain. The other (third to fifth) regions are yet undescribed.

### Conserved Sequences within the N-terminal Region

**Fig. 2.**
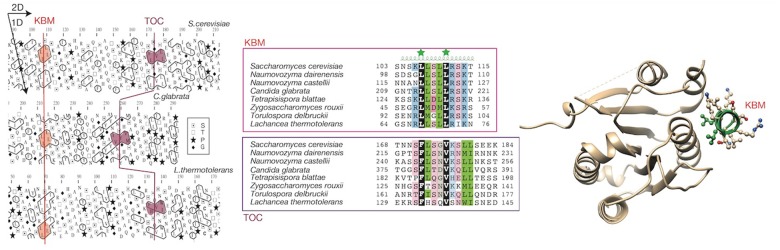
—The KBM–TOC region. Alignment of two conserved sequences defining the KBM and TOC motif. At left are shown the alignment of the 2D HCA plots of Sir4 from three species, as well as the corresponding 1D sequence alignments, extended to several other species. At right is shown the 3D structure of the recently solved KBM–KU complex (PDB 5Y59), with the two highly conserved leucine from KBM highlighted in green. On the HCA plots, the sequence is shown on a duplicated alpha helical net, on which the strong hydrophobic amino acids (V, I, L, M, F, Y, W) are contoured. These form clusters, which have been shown to mainly correspond to regular secondary structures (Gaboriaud et al. 1987; Callebaut et al. 1997). The way to read the sequence and the secondary structures are indicated with arrows. Special symbols are reported in the inset. In the alignment, positions conserved over the family are colored in green for hydrophobic amino acids (V, I, L, F, M, Y, W), in blue and pink for basic and acidic ones, in gray for loop forming residues (P, G, D, N, S). The UniProt identifiers of the sequences are reported in supplementary table S1, Supplementary Material online. No significant trace of KBM, moreover accompanied by a downstream TOC motif, could be found in the *Kluyveromyces lactis* and *Eremothecium* Sir4 sequences (both motifs presented in figure 3 of Chen et al. [2018] actually correspond to Asf2 sequences).

The few hydrophobic clusters of the N-terminal region have binary patterns (where 1 stands for any strong hydrophobic amino acid [V, I, L, M, F, Y, W] and 0 for other ones) typical of α-helices and β-strands, as deduced from our dictionary of hydrophobic clusters (Eudes et al. [2007]; supplementary fig. S1, Supplementary Material online). Most of them have limited lengths, except from nine clusters including four or more strong hydrophobic amino acids. Two of these last clusters (between aa 107 and 111 [binary code 11011] and aa 172 and 180 [binary code 110010011]), typical of α-helices, are detectable in Sir4 sequences from some species of the *Saccharomycetaceae* family, being, however, separated by sequences of variable length and embedded in predicted disordered regions (figs. 1 and 2). The first cluster is a KBM, which was recently observed in an α-helical structure in complex with KU (Chen et al. 2018) [PDB 5Y59]. The second cluster is a yet undescribed functional motif, which we called TOC for “second of TwO Clusters.” Of note is that these similarities could not be identified using standard search programs such as PSI-BLAST, due to the low sequence identities and the nonglobular character of the sequences. Instead, they were found by inspection of the conservation of HCA binary patterns. Although not detected here, we cannot exclude that KBM and TOC motifs are present, with degenerated sequences, in some members of the *Saccharomycetaceae* family, such as *K.lactis* and *Eremothecium cymbalariae*. No obvious sequence conservation was observed around the other hydrophobic clusters of the N-terminal half of Sir4.

### The SID and Relationship between Sir4 and Asf2

**Fig. 3.**
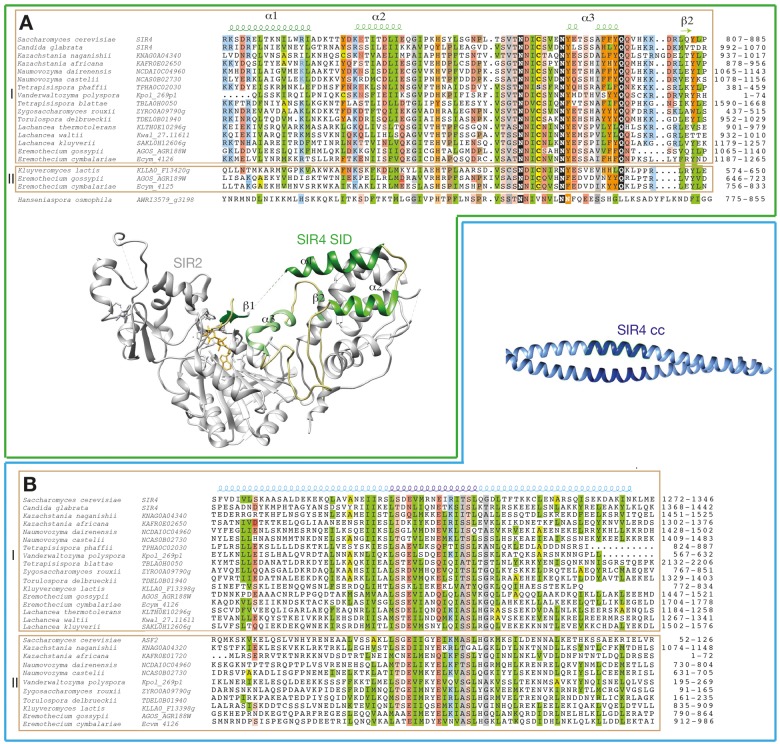
—The SID and coiled-coil (CC) of *S. cerevisiae* Sir4 and Asf2 and related proteins in the *Saccharomycetaceae* clade. These domains are analyzed within the two groups (I and II) of proteins (designated with the corresponding species and gene names), including *S. cerevisiae* Sir4 and Asf2 and defined according to the YGOB synteny and to the similarity relationships identified here. (*A*) Multiple alignment of the SID (except from the most variable N-terminal region, including strand β1). (*B*) Multiple alignment of the CC region. In the alignments, positions conserved over the family are colored in green for hydrophobic amino acids (V, I, L, F, M, Y, W), light green for amino acids that can substitute for hydrophobic amino acids in a context-dependent way (A, T, S, C), orange for aromatic amino acids (F, Y, W), gray for small amino acids (G, V, A, S, T), brown for P, yellow for the conserved C, blue for basic (K, R, H) amino acids, and pink for acidic (D, E) amino acids. Domain limits are reported at the end of the sequences, and the UniProt identifiers of the sequences are reported in supplementary table S1, Supplementary Material online. Between the alignments are reported the ribbon representations of the SID-Sir2 3D structure complex (PDB 4AIO) and of the Sir4 CC (PDB 1PL5). The corresponding regular secondary structures are reported up the alignments.

The second half of the Sir4 protein sequences contains seven regions that are predicted as foldable (see before, supplementary fig. S1, Supplementary Material online). The two first ones correspond to the SID, whose 3D structure has been solved in complex with Sir2 (Hsu et al. 2013) [PDB 4AIO]. In isolation, the Sir4 SID does not appear to have a stable structure but folds in contact with Sir2 through multiple sites (fig. 3*A*). One of these sites, accommodating Sir4 strand β1, corresponds to the first foldable segment, whereas the second foldable segment includes helices α1 to α3 and strand β2. The two segments are separated by a disordered sequence. SID is well conserved in the Sir4 proteins of the whole *Saccharomycetaceae* family (fig. 3*A*, top panel). Using PSI-BLAST with the *S. cerevisiae* SID sequence as query, we also found this domain in a hypothetical protein (g3198) from *Hanseniaspora osmophila* (UniProt A0A1E5R7W4), a member of the *Saccharomycodaceae* family, which is placed just nearby the *Saccharomycetaceae* family, according to recent phylogeny trees (Riley et al. 2016; Shen et al. 2016) (fig. 3*A*). This sequence shares the characteristics of the Sir4 family, with a significant proportion of disordered sequences which may undergo disorder-to-order transitions, and a C-terminal coiled-coil. Using this sequence (either the SID or the whole sequence) for further similarity searches did not lead to reveal any potential Sir4 sequences in more distant yeast species.

Of note in the *Saccharomycetaceae* family is that SID is found in two protein sequences of *Eremotheciumgossypii* (AGR188Wp and AGR189Wp), whose genes are contiguous (supplementary fig. S2, Supplementary Material online). Looking at corresponding contiguous genes also present in pre-whole genome duplication (WGD) (*K. lactis, Z. rouxii*, and *Torulospora delbruckii*) and post-WGD species (*N. castelli*, *N. dairensis, K. naganishii*, and *Vanderwaltozyma polyspora*) (supplementary fig. S2, Supplementary Material online), one can observe that all proteins syntenic to AGR188Wp (group I) except one possess an SID (green boxes) and a C-terminal coiled-coil (blue boxes), displaying the same architecture than the *S. cerevisiae* Sir4. In these species, a newly discovered H-BRCT domain (orange boxes, see below) is also present between the SID and coiled-coil region. The only exception is for *K. lactis* F13398p (syntenic to *E. gossypii* AGR188Wp), which lacks SID but maintains the H-BRCT domain and coiled-coil in similar positions to that observed in other species. In contrast, none of the products of the genes syntenic to *E. gossypii* AGR189Wp (group II) except one (*K. lactis* F13420p) possesses as AGR189Wp an SID. A conserved motif (blue boxes) was detected in this group II between these proteins and *S. cerevisiae* Asf2, which moreover share similarities with the Sir4 C-terminal coiled-coil (figs. 1 and 3*B*, supplementary fig. S2, Supplementary Material online). These observations of shared domains (SID, limited to two species, and coiled-coil) thus further support a common origin for Sir4 and Asf2 at the emergence of the *Saccharomycetaceae* family, likely stemming from a tandem gene duplication (Byrne and Wolfe 2005; Hickman et al. 2011; Gartenberg and Smith 2016). Asf2 is missing in some species (from the *Lachancea* clade and in *C.glabrata*). Asf2 from *S. cerevisiae* interacts with Sir3 and has an undefined role in silencing regulation (Le et al. 1997; Buchberger et al. 2008). We show here that, contrary to Sir4, Asf2 from *S. cerevisiae* is not required for telomere protection against fusions by NHEJ (supplementary fig. S3, Supplementary Material online). Interestingly, F13420p from *K. lactis*, which is syntenic to *E. gossypii* AGR189Wp and possesses an SID, was previously described as the Sir4 protein in *K. lactis*, because it complements the mating defect of a *S. cerevisiae sir4Δ* mutant and that its deletion in *K. lactis* derepresses the cryptic *HML-α1* gene (Aström and Rine 1998; Hickman and Rusche 2009). This suggests a separation of functions in *K. lactis*, with the protein bearing the SID holding the Sir2-dependent transcriptional repression activity and the other holding other functions that remain to be explored.

### A New H-BRCT Domain in the C-terminal Domain of Sir4

The second region, separated from SID by a large hinge (supplementary fig. S1, Supplementary Material online), includes at its C-terminal extremity the coiled-coil dimerization domain, whose structure has also been solved (Chang et al. 2003; Murphy et al. 2003) [PDB 1PL5 and 1NYH].

**Fig. 4.**
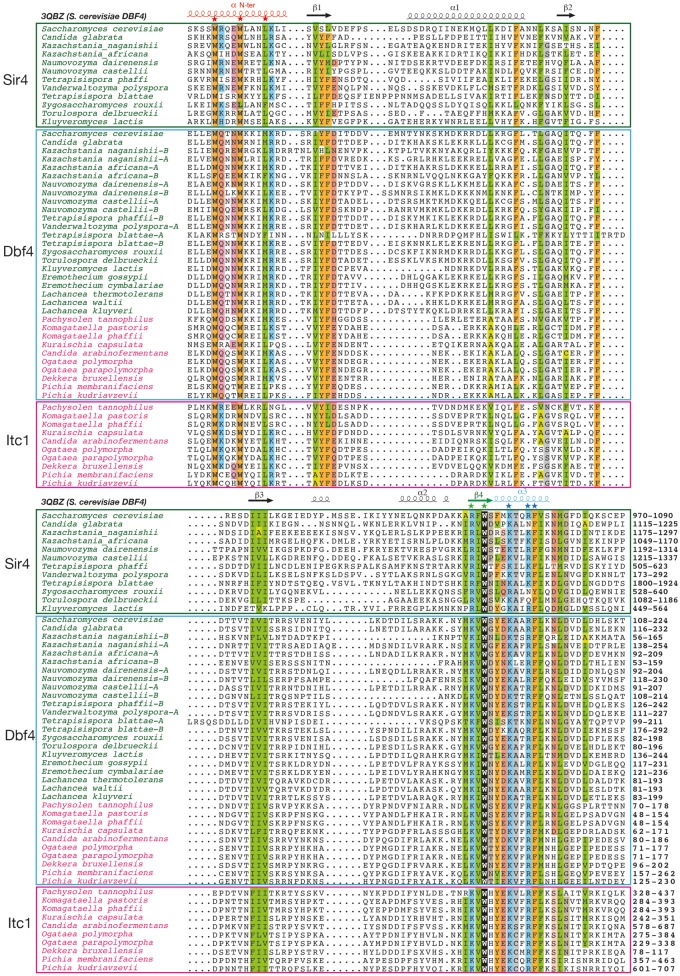
—The H-BRCT domains of *S. cerevisiae* Sir4 and related proteins of the *Saccharomycetaceae* clade and of Itc1 from the methylotrophs clade compared with those of Dbf4. Multiple alignment of the H-BRCT domain sequences. Sequences related to *S. cerevisiae* Sir4 belong to the group I sequences, as described in supplementary table S1, Supplementary Material online. Regular secondary structures based on the experimental 3D structure of the *S. cerevisiae* Dbf4 H-BRCT domain (PDB 3QBZ; Matthews et al. 2012) are reported above the alignment. Positions conserved over the entire H-BRCT family are colored in green for hydrophobic amino acids (V, I, L, F, M, Y, W), light green for amino acids that can substitute for hydrophobic amino acids in a context-dependent way (A, T, S), orange for aromatic amino acids (F, Y, W), yellow for loop-forming amino acids (P, G, D, N, S) and related amino acids (E, T), blue for basic (K, R, H) amino acids, and pink for acidic (D, E) amino acids. Highly conserved amino acids reported on the representation of the 3D structure of yeast Dbf4 H-BRCT domain (fig. 5) are depicted with stars. Domain limits are reported at the end of the sequences, and the UniProt identifiers of the sequences are reported in supplementary table S1, Supplementary Material online.

Searching the NCBI *nr* database using the sequence of the region encompassing aa 970–1197 (region preceding the extended coiled-coil region) as query using PSI-BLAST revealed significant similarities on its first part, encompassing a hundred amino acids (aa 970–1087). These are observed with Sir4 protein sequences of all the species of the *Saccharomycetaceae* family, up to *K.lactis* (fig. 4, upper panel), with the exception of Sir4 proteins from the *Eremothecium* and *Lachancea* clades (*L. kluyverii, L. thermotolerans*). From the second PSI-BLAST iteration onward, significant similarities then appeared between this Sir4 conserved domain and the H-BRCT domain of yeast Dbf4, whose experimental 3D structure has been solved (Matthews et al. 2012) [PDB 3QBZ] (fig. 4, middle panel).

Examination of the HCA plots of the Sir4 sequences from the *Lachancea* clade suggests that the H-BRCT region is likely present in these proteins, as supported by some conserved clusters and some sequence motifs, but should be degenerated relatively to well-conserved amino acids (supplementary figs. S4*A* and S5, Supplementary Material online). In particular, two highly conserved tryptophan residues at the beginning of the H-BRCT domain are not present in the *Lachancea* sequences. Such hypothesis is also supported by similarities reported, however, with nonsignificant E-values in the PSI-BLAST results. In contrast, no trace of an H-BRCT domain could be found in the *Eremothecium* Sir4 sequences, although some hydrophobic clusters are still present between the SID and the coiled-coil region (supplementary fig. S4*A*, Supplementary Material online). No H-BRCT trace could be highlighted in the *Hanseniaspora* Sir4-like protein. The Sir4 sequences downstream the H-BRCT domains (i.e., between the H-BRCT domain and the coiled-coil region) show no obvious similarities with any other protein sequences.

### An H-BRCT Domain Is Also Found in Itc1 Proteins from the Methyltrophs Clade

An H-BRCT domain is also retrieved in all the Itc1 proteins from the methylotrophs clade (fig. 4, bottom panel and supplementary fig. S4*B*, Supplementary Material online), between a conserved, yet unknown domain (that we named downWAC) downstream of a WAC domain (after WSTF/Acf1/cbp146 [Ito et al. 1999]) and a DDT domain (after the better-characterized DNA-binding homeobox-containing proteins and the different transcription and chromatin remodeling factors in which it is found [Doerks et al. 2001]). This domain is not present in the Itc1 proteins of the *Saccharomycetaceae* clade, the corresponding sequence being shorter and generally disordered (supplementary fig. S4*B*, Supplementary Material online). It is also not retrieved in *Ascoidea rubescens* Itc1, an observation which may support a placement of this species outside the methylotrophs clade, according to the comments and trees reported in recent studies (Riley et al. 2016; Shen et al. 2016).

### Conserved Sequence Features of H-BRCT Domains

**Fig. 5.**
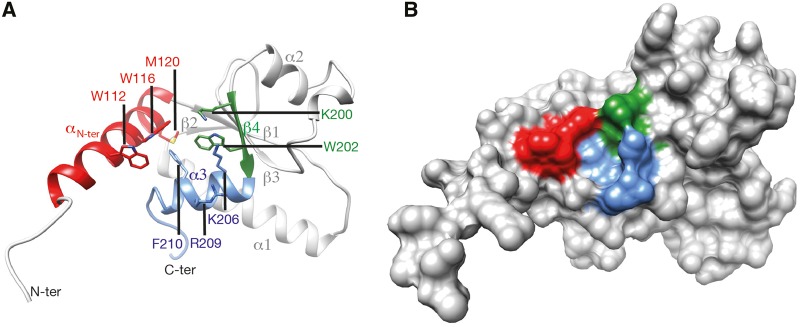
—The H-BRCT domain 3D structure. Experimental 3D structure of the H-BRCT domain of *S. cerevisiae* Dbf4 (PDB 3QBZ; Matthews et al. 2012), on which are highlighted the highly conserved positions depicted with stars in figure 5. (*A*) Ribbon representation. (*B*) Solvent accessible surface.

The enlargement of the H-BRCT family to Sir4 and Itc1 allowed to identify the few conserved amino acids which may play a key functional role. The multiple sequence alignment of the Sir4, Dbf4, and Itc1 H-BRCT domains is shown in figure 4, together with the secondary structures observed in the *S. cerevisiae* Dbf4 H-BRCT 3D structure [PDB 3QBZ]. The corresponding 3D structure is reported in figure 5 (surface and ribbon representations), on which the highly conserved amino acids (stars in fig. 4) are highlighted. The Dbf4 H-BRCT domain consists in a bona fide BRCT core, comprising four β-strands and three α-helices, accompanied by a long N-terminal helix (α N-ter) that participates in the core of the domain. The concave surface defined by α1, β4, and α4, important for the binding of Dbf4 with Rad53 (Matthews et al. 2012), concentrates most of the amino acids that are conserved between Sir4, Itc1, and Dbf4 (fig. 5). Mutation of W202 (star in strand ß4) in yeast Dbf4 has been shown to cause increased sensitivity to DNA-damaging agents and hydroxyurea (Gabrielse et al. 2006) and to lose the interaction with Rad53 (Matthews et al. 2012). This interaction is also lost with mutations of W116 and M120 (stars in helix α Nter), as well in constructs with the L109A/W112D and W116D/M120A double mutations (Matthews et al. 2012). The importance of these amino acids has been suggested to be due to their participation in the hydrophobic core of the H-BRCT domain. However, one can also note that the four highly conserved aromatic amino acids (W202, W112, W116, and F210 in the *S. cerevisiae* Dbf4 sequence) form a putative binding cage (with F210 at the bottom, fig. 5), suggesting a possible role in the interaction.

The region encompassing the H-BRCT domain mediates the interaction of *S. cerevisiae* Sir4 with both peripheral membrane anchor Esc1 (Ansari and Gartenberg 1997; Andrulis et al. 2002; Taddei et al. 2004) and the Ty5 integrase (Zhu et al. 2003; Kvaratskhelia et al. 2014). Interestingly, Sir4 amino acids W974 and R975 (helix α Nter) have been shown to be critical for the interaction with Ty5, W974 occupying in Sir4 a position in Dbf4 (W112) that have been shown to be critical for interaction with Rad53 (Matthews et al. 2012).

To assess Sir4 H-BRCT domain function in *S. cerevisiae*, we mutated the conserved tryptophan residues W974 and W978 into alanine (*sir4-AA*; supplementary fig. S6*A*, Supplementary Material online). Integrated at the endogenous locus, the mutant allele impacts protein stability (moderately in exponentially growing cells, more severely in stationary cells) and causes a silencing defect at *HML* (supplementary fig. S6*B* and *C*, Supplementary Material online). This defect is suppressed by the addition of one or a few extra copies of *sir4-AA* (supplementary fig. S6*C*, Supplementary Material online), indicating that the two mutated residues are not essential for transcriptional silencing when Sir4 protein level is closer to wild-type level. In addition, these two residues are required neither for the protection of *HML* against HO cleavage nor for the repair of HO-induced double-strand break at *MAT* (supplementary fig. S6*D*, Supplementary Material online). These results do not support an essential role for Sir4 H-BRCT domain in transcriptional silencing, protection from HO-induced cutting and mating-type switching. However, they do not rule out a contributive role.

### Identification of Conserved Motifs in Esc1

A clue for Sir4 H-BRCT domain function may come from its inclusion within the previously defined PAD of Sir4 (aa 960–1150), which interacts with the nuclear periphery protein Esc1 (Ansari and Gartenberg 1997; Andrulis et al. 2002; Taddei et al. 2004). Esc1 is a poorly defined protein. In *S. cerevisiae*, it anchors telomere at the nuclear periphery through its association with Sir4. This Esc1-dependent peripheral anchoring is a facultative contributor to transcriptional silencing (Andrulis et al. 2002; Taddei et al. 2004; Meister and Taddei 2013) and could be the function of Sir4 H-BRCT domain. Contrary to Sir4, Esc1 is not required for telomere protection against fusions by NHEJ (supplementary fig. S7, Supplementary Material online).

**Fig. 6.**
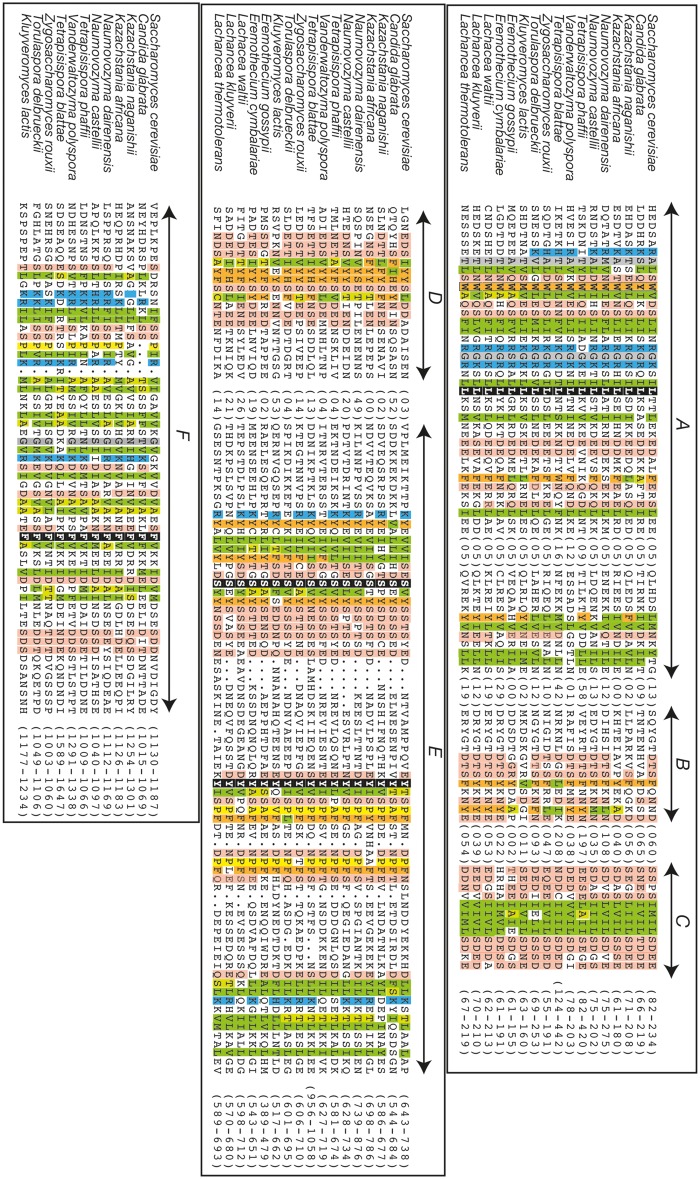
—The Esc1 conserved motifs. Multiple alignment of the Esc1 conserved motifs (*A*–*E*). In the alignments, positions conserved over the family are colored in green for hydrophobic amino acids (V, I, L, F, M, Y, W), light green for amino acids that can substitute for hydrophobic amino acids in a context-dependent way (A, T, S, C), orange for aromatic amino acids (F, Y, W), gray for small amino acids (G, V, A, S, T), yellow for P, blue for basic (K, R, H) amino acids, and pink for acidic (D, E, Q, N, S, T) amino acids. Domain limits (in brackets) are reported at the end of the sequences, and the UniProt identifiers of the sequences are reported in supplementary table S1, Supplementary Material online.

As Esc1 structure was unexplored, we searched for foldable regions in this large protein (1,658 amino acids). We identified six motifs conserved between Esc1 from the different species (Motifs Esc1-A to Esc1-F; figs. 1 and figs. 6, supplementary fig. S8, Supplementary Material online). Motif Esc1-A is predicted to form a globular domain, mainly made of α-helices. Motif Esc1-B appears less conserved in some sequences (such as *C.glabrata* and *K.lactis*), whereas motif Esc1-C, consisting of a β-strand motif followed by a serine and acidic sequences, evokes the possibility of a conserved SUMO-interacting motif (SIM) (Kerscher 2007). Note that this motif could be highlighted in some sequences (such as *Tetrapisispora*) only by visual inspection of the HCA plot, as it is short and separated from other conserved motifs by sequences of variable length (supplementary fig. S8, Supplementary Material online). Interestingly, the SUMO E3 ligase Siz2 SUMOylates Sir4 (in particular at position K1128, i.e., within the PAD downstream of the H-BCRT domain) and perinuclear anchoring through Sir4 also requires Siz2 (Ferreira et al. 2011; Kueng et al. 2012). In addition, Sir4, Esc1, and Siz2 are part of a larger complex with a subset of nucleoporins (Lapetina et al. 2017). The SIM within motif Esc1-C might thus contribute to the regulation of this complex. The two following conserved motifs (Esc1-D and Esc1-E) are found in all species of the *Saccharomycetaceae* family examined, including in particular some highly conserved aromatic amino acids. Finally, a conserved motif (Esc1-F), likely adopting a helical structure, is found in the C-terminal part of Esc1 proteins from the *Saccharomycetaceae* family, except from the *Lachancea* and *Eremothecium* (fig. 1). Note that the more divergent motif F of the *C.glabrata* Esc1 was found only after examination of the HCA plot (supplementary fig. S8, Supplementary Material online). The apparent absence of motif Esc1-F in species lacking a functional H-BRCT in Sir4 raises the interesting possibility that these two domains coevolve (although we cannot exclude that such a divergent motif is present but undetected yet in the *Lachancea* and *Eremothecium* sequences). Motif Esc1-F is unlikely to interact with Sir4 because the region sufficient for Esc1-Sir4 interaction is situated more downstream in Esc1 (aa 1395–1551; Andrulis et al. 2002). No conserved motif could, however, be highlighted in this region. No obvious similarity could also be highlighted with known 3D structures for the different motifs using HHpred and Phyre.

## Discussion

Sir4 is an essential scaffold for Sir complex assembly and the less understood component of yeast heterochromatin. Here, we find in Sir4 a functional Dbf4-related H-BRCT domain positioned within the PAD between the SID and the C-terminal coiled-coil. We predict a new functional region called TOC in Sir4 N-terminal region. Additionally, we identify six new conserved motifs in Sir4-interacting partner and nuclear-envelope associated protein Esc1. Finally, we find in species of the Sir4-free methylotrophs clade a related H-BRCT domain within a distinct protein, Itc1, a component of the chromatin remodeler ISWI2.

The molecular functions of these newly identified domains remain to be deciphered. As the TOC region is within an Sir4 region interacting with KU (Yku80), Rap1, and DNA (Kueng et al. 2012; Gartenberg and Smith 2016; Chen et al. 2018), it may contribute to these interactions. Sir4 H-BRCT interacts with Esc1 and therefore is likely to participate in heterochromatin anchoring at the nuclear periphery through this interaction, whose functional significance is to favor the establishment of a repressive domain within the nucleus (Meister and Taddei 2013).

Interestingly, on the one hand H-BRCT domains are missing in Sir4 from *E. gossypii* and *E. cymbalariae* (Eremothecium clade) and degenerated in the genus *Lachancea*. A commonality of these species is the absence of both Esc1 motif Esc1-F and the HO endonuclease (Butler et al. 2004; Wendland and Walther 2011; Agier et al. 2013). How they switch mating type is unknown. On the other hand, functional H-BRCT domain and Esc1 motif F are present in the genus *K.lactis*, also lacking HO, but where domesticated transposases (α3/Kat1) create double-strand DNA breaks at the *MAT* locus to induce mating-type switching (Barsoum et al. 2010; Rajaei et al. 2014). Thus, the presence of an H-BRCT domain in Sir4 and a motif F in Esc1 seems to correlate with how mating-type switching is induced. This observation suggests an unsuspected link between Esc1 and the recombination of *MAT* with the silent donors. Several models can be envisaged. For instance, the anchoring of silent mating-type cassettes to the nuclear periphery may contribute to homologous recombination within a heterochromatic template or to the maintenance of transcriptional silencing during and after repair, hypotheses remaining to be addressed. Sir4 and Esc1 may also act more indirectly by regulating checkpoint activation or replication origin firing.

The last hypotheses are relevant because DNA damage checkpoint is blind to mating-type switching (Pellicioli et al. 2001) and Sir4-associated replication origins are inefficient or inactive (Dubey et al. 1991; Palacios DeBeer and Fox 1999; Rivier et al. 1999; Vujcic et al. 1999; Sharma et al. 2001; Zappulla et al. 2002; Palacios DeBeer et al. 2003). These hypotheses would also link Sir4 H-BRCT function with the function of the H-BRCT originally found in the ubiquitous Dbf4, which is to regulate origin firing through an interaction with the checkpoint kinase Rad53 (Gabrielse et al. 2006; Lopez-Mosqueda et al. 2010; Zegerman and Diffley 2010; Matthews et al. 2012, 2014; Chen et al. 2013) (reviewed by Diffley [2010]). Remarkably, Sir3 originates from the ORC subunit Orc1 through the last WGD (Byrne and Wolfe 2005; Gartenberg and Smith 2016). In *K. lactis*, a pre-WGD species of the *Saccharomycetaceae* family, Orc1 is required for heterochromatin and a likely partner of Sir4 (Hickman and Rusche 2010; Hickman et al. 2011; Hanner and Rusche 2017). The finding reported here adds a novel example suggesting that yeast heterochromatin evolved in part from the replication initiation machinery. The significance of this origin remains to be understood.

Itc1 is a regulatory subunit of the ISWI2 chromatin remodeler. In *S. cerevisiae*, it contributes to the transcriptional repression of mating-type-specific genes (Fazzio et al. 2001; Ruiz et al. 2003). Addressing the role of Itc1 and its H-BRCT domain in methylotrophs could be an entry point to decipher the mechanisms behind the two *MAT*-like-loci system of these yeasts apparently lacking Sir4 and HP1. Together, our findings show the importance of exploring the dark proteome and provide new insights on the evolution of yeast heterochromatin and life cycle.

## Supplementary Material

Supplementary data are available at *Genome Biology and Evolution* online.

## Supplementary Material

Supplementary Data

## Notes

**Data availaibility**: All the analyzed sequences (supplementary table S1, Supplementary Material online) are publicly available from UniProt. The 3D structure coordinates are publicly available from RCSB Protein Data Bank.
