# Supplementary material for: Discovery and Evolution of New Domains in Yeast Heterochromatin Factor Sir4 and Its Partner Esc1
Source: Genome Biol Evol. 2019 Jan 22;11(2):572–85. doi: 10.1093/gbe/evz010 (PMC6394760; doi:10.1093/gbe/evz010)
Supplement: Supplementary Data [file evz010_supp.pdf]

Discovery and Evolution of New Domains in Yeast Heterochromatin Factor Sir4 and its Partner Esc1

SUPPLEMENTARY DATA

Guilhem Faure *1,3, Kévin Jézéquel *2, Florian Roisné-Hamelin *2, Tristan Bitard-Feildel 1,4, Alexis Lamiable 1, Stéphane Marcand #2 and Isabelle Callebaut #1

1. Sorbonne Université, Muséum National d'Histoire Naturelle, UMR CNRS 7590, IRD, Institut de Minéralogie, de Physique des Matériaux et de Cosmochimie, IMPMC, 75005 Paris, France

2. Institut de Biologie François Jacob, IRCM/SIGRR/LTR, INSERM UMR 967, CEA Paris-Saclay, France

3. *Present address:* National Center for Biotechnology Information, National Library of Medicine, National Institutes of Health, Bethesda, MD 20894, USA

4. *Present address:* Sorbonne Université, UMR CNRS 7238, IBPS, Laboratoire de Biologie Computationnelle et Quantitative (LCQB), 75005 Paris, France

* Equal contributions

# Equal contributions and corresponding authors:

isabelle.callebaut@upmc.fr

stephane.marcand@cea.fr

		**I** (UniProt / *gene*)	**II** (UniProt / *gene*)	**Dbf4** (UniProt)	**Itc1** (UniProt)	**Esc1** (UniProt)
SACCHAROMYCETACEAE	*Saccharomyces cerevisiae*	**P11978 / *SIR4***	**P32448 /*ASF2***	P32325	P53125	Q03661
	Candida glabrata	Q6FM33 / *SIR4*	-	Q6FV57	Q6FRF3	Q6FUF1
	Kazachstania naganishii	J7S2D5 / *KNAG0A04340*	J7REX1 / *KNAG0A04320*	J7S2Y3, J7S8G4	J7S8C9	J7SAL8
	Kazachstania africana	H2AVL7 / *KAFR0E02650*	H2AZ26 / *KAFR0E01720*	H2AYY2, H2ARM9	H2B1U3	H2AQH5
	Naumovozyma dairenensis	G0W8P4 / *NDAI0C04960*	G0W8P5 (A) / *NDAI0C04970*	G0W4H1(A), G0W521(B)	G0WFU3(A), G0WCG0(B)	G0WHN1
	Naumovozyma castellii	G0VBN1 / *NCAS0B02730*	G0VBN0 (A) / *NCAS0B02720*	G0V836(A), G0VJY1(B)	G0V5C3(A), G0VEJ8(B)	G0VC82
	Tetrapisispora phaffii	G8BRI2 / *TPHA0C02030*	-	G8C0I6	G8BYX7	G8BVP5
	Vanderwaltozyma polyspora	A7TT32 / *Kpol_269p1*	A7TT33 / *Kpol_269p1*	A7TL67	A7TE72	A7TRH4
	Tetrapisispora blattae	I2H7J6 / *TBLA0H0050*	-	I2H913, I2H7B0	I2GV58	I2GYI5
	Zygosacharomyces rouxii	C5DQ98 / *ZYRO0A09790g*	C5DQ99 / *ZYRO0A09812g*	C5E0G9	C5E4M3	C5DPU1
	Torulospora delbruckii	G8ZNX9 / *TDEL0B01940*	G8ZNX8 / *TDEL0B01930*	G8ZTG8	G8ZVZ1	G8ZPF8
	Kluyveromyces lactis	Q6CK56 / *KLLA0_F13398g*	Q6CK55 / *KLLA0_F13420g*	Q6CKD0	Q6CWR5	Q6CU74
	Eremothecium gossypii	Q74ZW1 / *AGOS_AGR188W*	Q74ZW0 / *AGOS_AGR189W*	Q750Z2	Q755D5	Q756B4
	Eremothecium cymbalariae	G8JT52 / *Ecym_4126*	G8JT51 / *Ecym_4125*	G8JNV6	G8JP27	G8JVE7
	Lachancea waltii	*Kwal_27.11611*	-	*Kwal_27.10452*	*Kwal_56.24134*	*Kwal_26.9080*
	Lachancea kluyverii	*SAKL0H12606g*	-	*SAKL0D02574g*	*SAKL0A04004g*	*SAKL0H05104g*
	Lachancea thermotolerans	C5DI75 / *KLTH0E10296g*	-	C5DDY7	C5E2C1	C5DF68
METHYLOTROPHS	*Pachysolen tannophilus*	-	-	A0A1E4TQA9	A0A1E4TZW6	
	*Komagataella pastoris**	-	-	A0A1B2J8P9	A0A1B2JHS7	
	*Komagataella phaffii**	-	-	C4QV30	F2QTP2	
	Kuraishia capsulata	-	-	W6MM48	W6MQ02	-
	Candida arabinofermentans			A0A1E4T408	A0A1E4SUV7	
	Ogataea polymorpha	-	-	A0A1B7SCH7	A0A1B7SLG9	-
	*Ogataea parapolymorpha***	-	-	W1QIK4	W1QA82	-
	Dekkera bruxellensis ***	-	-	I2K3X6	I2K0S1	-
	Pichia membranifaciens	-	-	A0A1E3NMD2	A0A1E3NF40	-
	Pichia kudriavzevii	-	-	A0A099NWV2	A0A1V2LRF5	-

* formely called *Pichia pastoris,* ** formerly called *Hansenula parapolymorpha,* *** formerly called *Brettanomyces bruxellensis*

Supplementary Table S1: UniProt accession of the protein sequences reported in this study, except for those of *Lachancea waltii* and *Lachancea kluyverii*, which were directly extracted from genome data**.** The corresponding gene names (italics) were also given for groups I and II, which were defined according to the synteny described in YGOB and to the similarity relationships identified here (see text). A and B stand for the track considered, according to YGOB (http:// http://ygob.ucd.ie/).

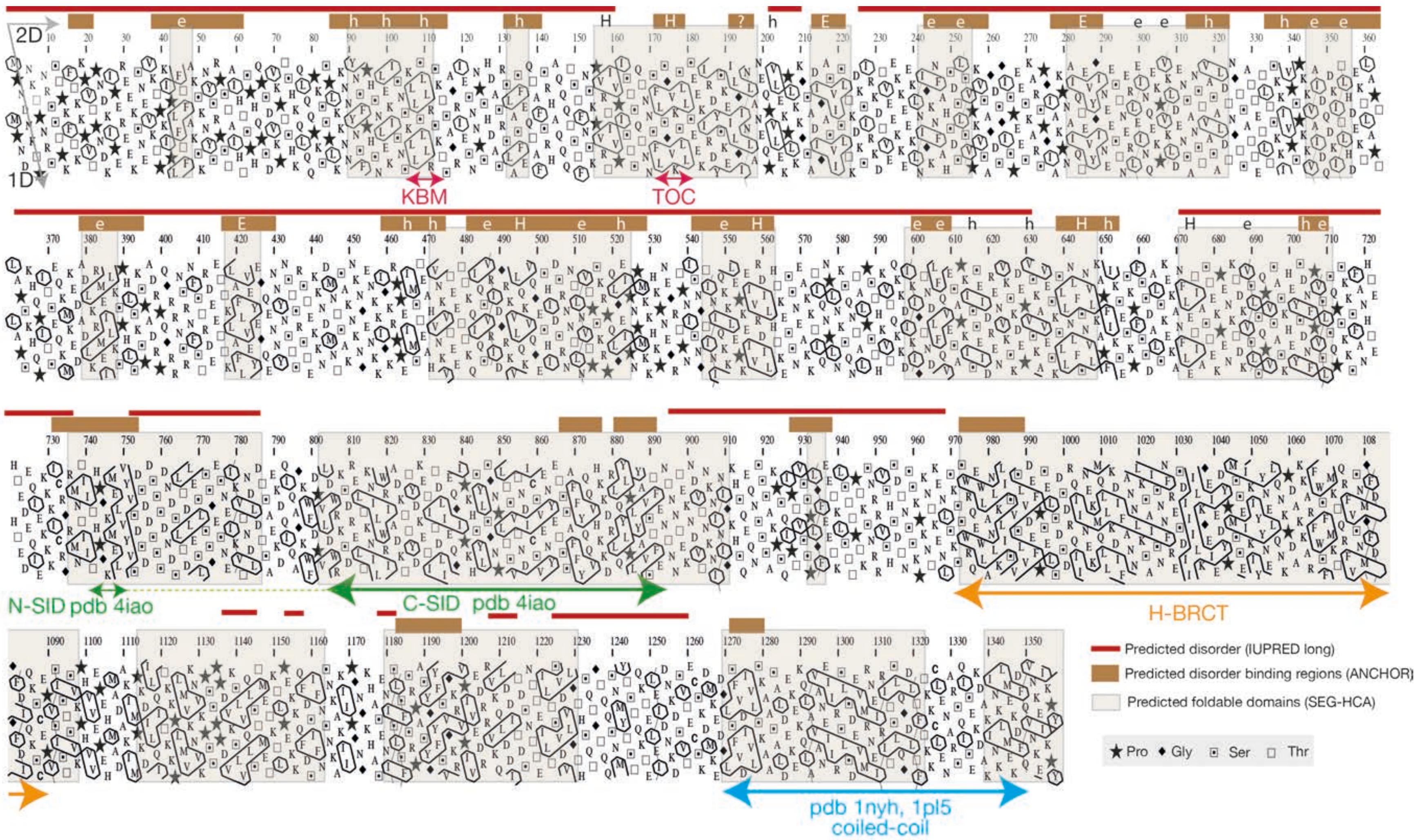

Supplementary Figure S1:

HCA plot of the *S. cerevisiae* Sir4 sequence. Briefly, the sequence is shown on a duplicated alpha helical net, on which the strong hydrophobic amino acids (V, I, L, M, F, Y, W) are contoured. These form clusters, which have been shown to mainly correspond to regular secondary structures (Gaboriaud et al. 1987; Callebaut et al. 1997). The way to read the sequence (1D) and the secondary structures (2D) are indicated with arrows. Special symbols are used for four amino acids (P,G,S,T), according to their particular structural behavior. Predictions made by the disorder predictor IUPRED are reported (Dosztányi et al. 2005a; Dosztányi et al. 2005b), as well as those from the associated ANCHOR program (binding regions within disordered segments) (Dosztányi et al. 2009; Mészáros et al. 2009). The predictions of foldable domains (described as regions with high density in hydrophobic clusters), as made by SEG-HCA (Faure and Callebaut 2013), are boxed and shaded grey on the HCA plot. Finally, the positions of known 3D structures are also reported on the HCA plot, as well as the position of the H-BRCT domain delineated here. The secondary structure affinities of hydrophobic clusters included in the N-terminal region are reported up to the plot (H/h: strong and weak affinity for the α–helical state, E/e: strong and weak affinity for the β–strand state), These were deduced from experimental databases (HCDB v2), updated from (Eudes et al. 2007).

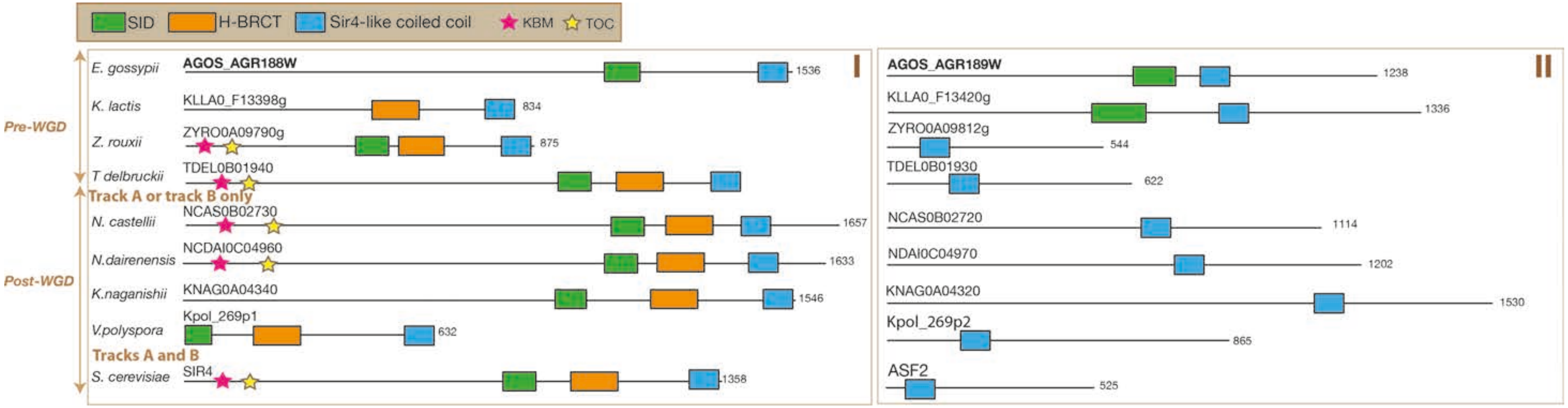

Supplementary Figure S2:
Architecture of proteins whose corresponding genes are contiguous in the same track (according to YGDB), compared to the *S. cerevisiae* SIR4 (group I) and ASF2 (group II) architectures.

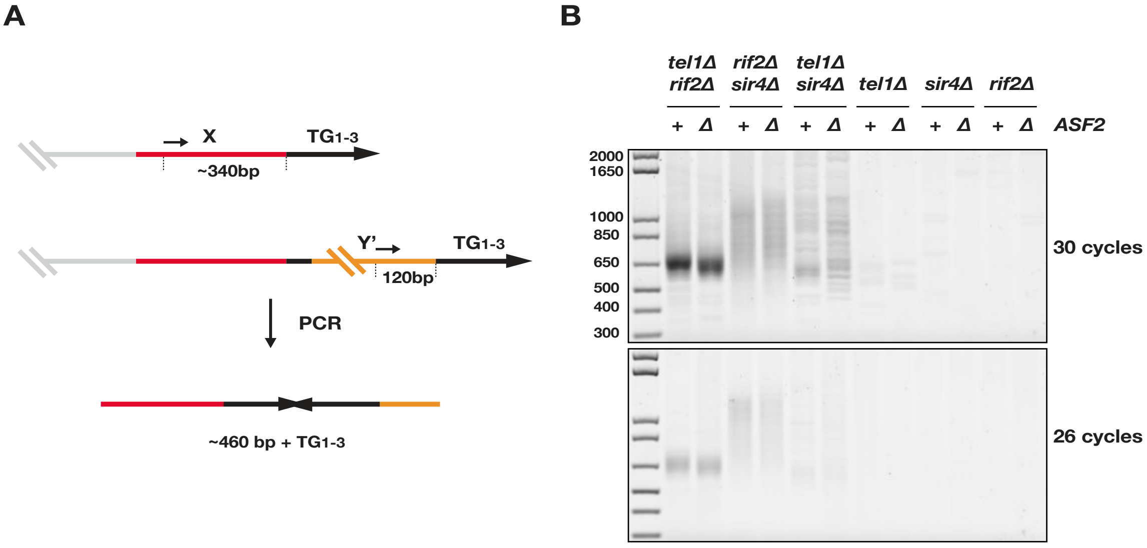

Supplementary Figure S3:

Asf2 is not required for NHEJ inhibition at telomeres in *S. cerevisiae*. A) To address a defect in NHEJ inhibition at telomeres in cells lacking Asf2, we looked at the appearance of telomere fusions, which can to some extent be amplified by PCR. All *S. cerevisiae* chromosome ends display a conserved X subtelomeric element. About half the chromosome ends contain one or several Y' subtelomeric elements inserted between the X element and the telomere. We used two primers to amplify fusions between Y' and X-only telomeres. Telomere length being heterogeneous, amplified telomere fusions appear as a smeared signal. The length of the PCR products indicates the length of telomeric repeats in the fusions. **B)** Cells were grown exponentially in rich medium and allowed to reach stationary phase in 6 days. Fusions between X and Y' telomeres were amplified by PCR with 30 and 26 cycles (as described in (Lescasse et al. 2013)). Rarer fusions are amplified as discrete bands. Asf2 loss has no impact on telomere fusion frequency in contexts where the loss of Tel1, Rif2 or Sir4 weaken telomere protection (Tel1 loss by shortening telomeres, Rif2 and Sir4 loss by eliminating two parallel pathways inhibiting NHEJ (Marcand et al. 2008)). This result rules out a role for Asf2 similar to Sir4 in telomere protection, at least in *S. cerevisiae*.

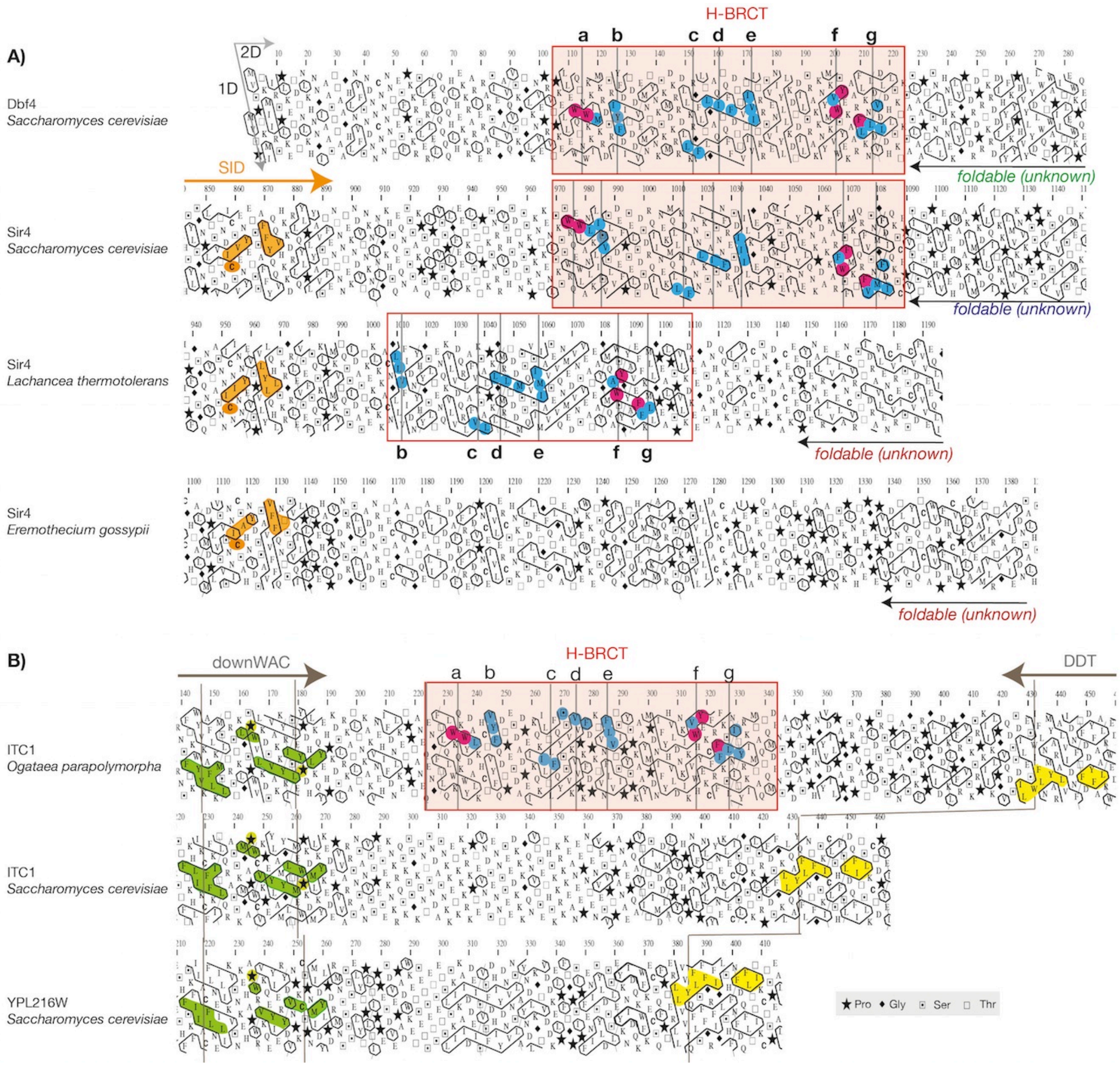

Supplementary Figure S4:

Presence/absence of H-BRCT domains in Dbf4, Sir4 (Saccharomycetaceae clade) and Itc1 (methylotroph clade) sequences, as highlighting using HCA. Principles of HCA plots are described in Supplementary Figure 1. Conserved clusters are reported in blue (H-BRCT) and in green, orange and yellow (other domains), whereas highly conserved amino acids of the H-BRCT domain are colored in pink. **A)** Sequences of the H-BRCT domains of Sir4 from *S. cerevisiae* and *L. thermotolerans* (*Saccharomycetaceae* clade), compared to the H-BRCT domain of *S. cerevisiae* Dbf4p. The *L. thermotolerans* H-BRCT domain lacks conserved motifs of canonical H-BRCT domains. The *E. gossypii* Sir4 sequence is also shown, in order to highlight the presence of a globular domain, however with no detectable similarity with the H-BRCT domain. **B)** Sequences of Itc1 from *O. parapolymorpha* (methylotrophs clade) compared to the two syntenic orthologs of *S. cerevisiae*, lacking the H-BRCT domain.

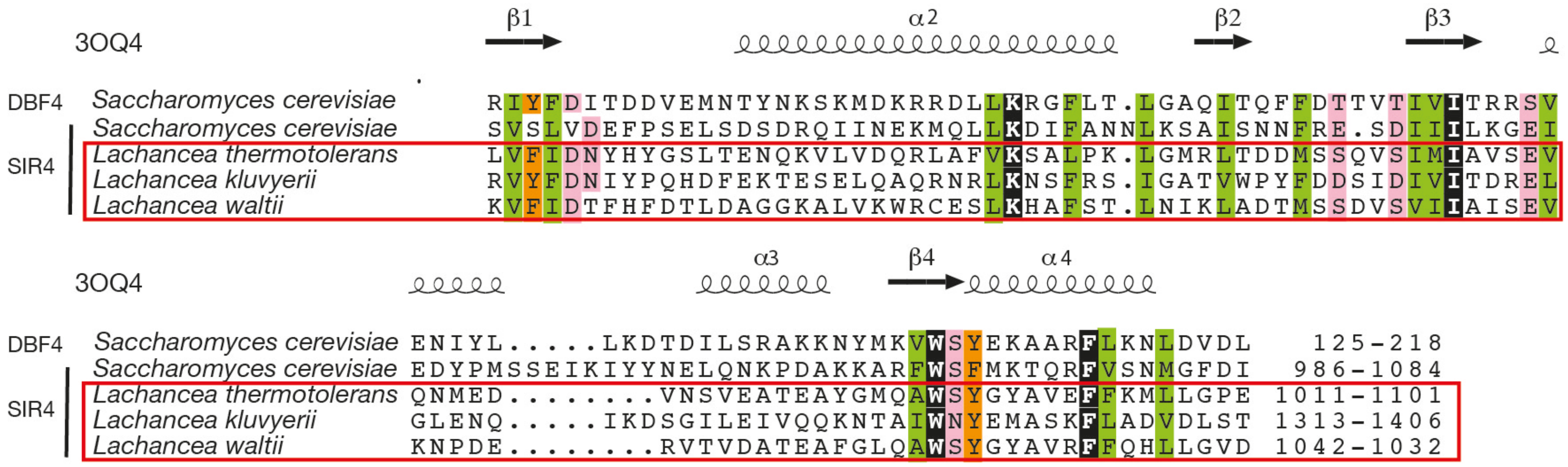

Supplementary Figure S5:
Alignment of the H-BRCT sequences from *Lachancea* Sir4 proteins, compared to that of *S. cerevisiae.*
The conserved motifs of the N-terminal α-helix, including the two highly conserved tryptophane, are not found in the *Lachancea* sequences.

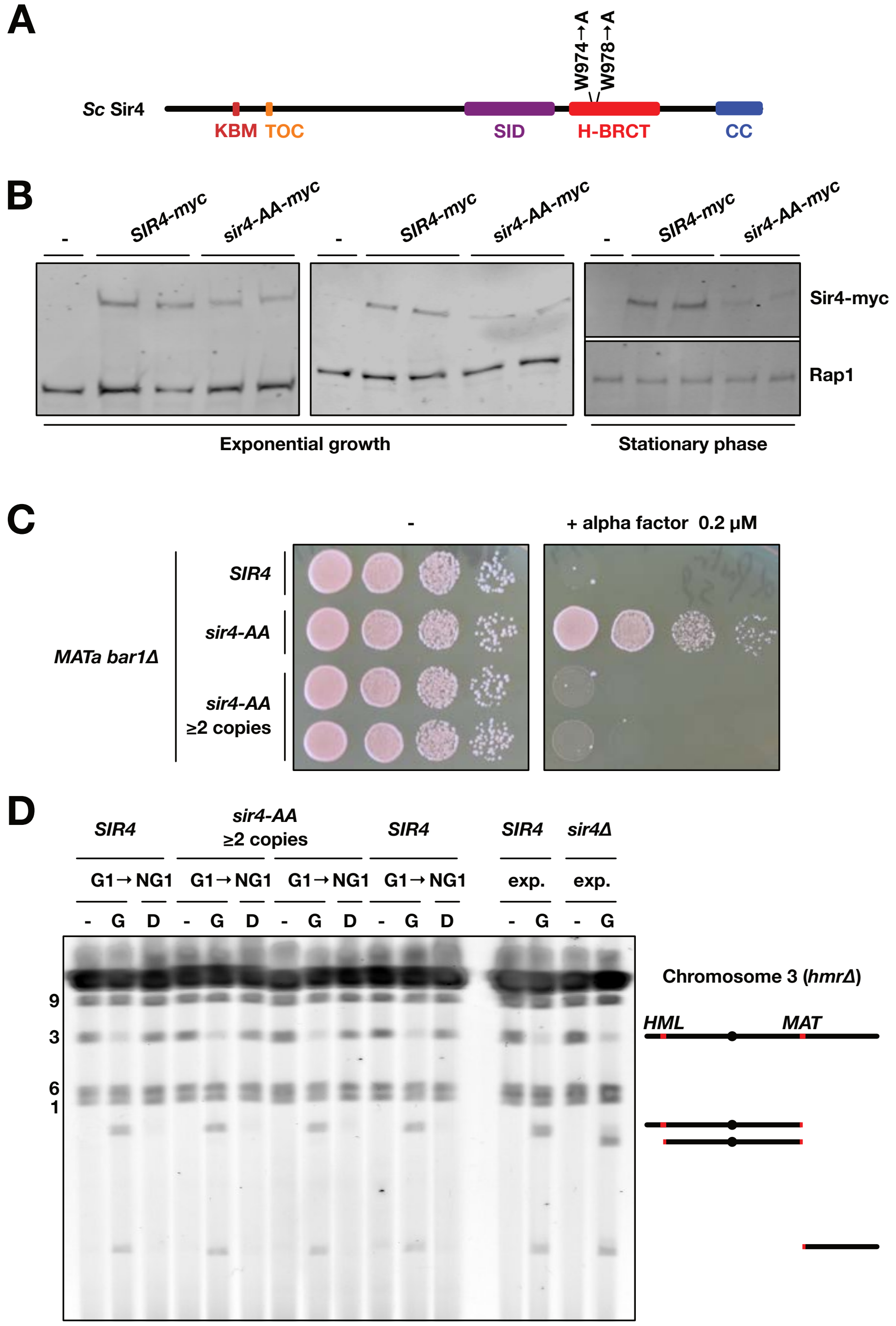

Supplementary Figure S6:

Sir4 H-BRCT conserved residues W974 and W978 are not required for *HML* silencing nor for HO-induced mating type switching in *S. cerevisiae*. A) Schematic representation of *S. cerevisiae* Sir4. In the *sir4-AA* allele, conserved tryptophan residues 974 and 978 are mutated into alanine. **B)** The *sir4-AA* allele integrated at the endogenous locus impacts Sir4 stability in exponential and stationary phases. Cells with an untagged WT *SIR4* (-), a myc^{13} tagged WT *SIR4* (*SIR4-myc*) and a myc^{13} tagged mutant *sir4-AA* (*sir4-AA-myc*) were grown exponentially in rich medium or allowed to reach stationary phase. Proteins extracted by urea were analyzed by western blot. Membranes were probed with antibodies against the myc epitope, and the Rap1

protein as a loading control. Each lane from an independent culture and extraction. **C)** The *sir4-AA* caused a silencing defect at *HML* that is suppressed by increased *sir4-AA* copy number. *MAT**a*** cells lacking the alpha-factor-specific Bar1 protease and carrying either a WT *SIR4* allele (*SIR4*), a single copy *sir4-AA* allele (*sir4-AA*) or multiple copies of the *sir4-AA* allele (*sir4-AA* ≥2 copies, tandem integration of a pRS406-sir4-AA plasmid within *sir4-AA* at the endogenous locus) were spotted on rich medium with or without alpha factor and grown 2 days at 30°C. 10-fold successive dilutions. Transcription of *HML* in single-copy *sir4-AA* cells allows these cells to partially escape the growth inhibition induced by alpha factor. **D)** Efficient HO-induced mating type switching in *sir4-AA* cells. *MAT**a*** cells lacking the alpha-factor specific Bar1 protease, transformed with a pRS314-pGAL1-HO plasmid and carrying either a WT *SIR4* allele (*SIR4*) or multiple copies of the *sir4-AA* allele (*sir4-AA* ≥2 copies) at the endogenous locus were grown in glycerol-lactate medium lacking tryptophan, synchronized in G1 with alpha-factor (G1 -, HO unexpressed), exposed to galactose for 40 minutes (G1 G, HO induced) and then released from G1 in glucose rich medium to be blocked again in the next G1 with alpha-factor (NG1 D, HO repressed). Chromosomes were separated by PFGE and labelled with Gel Red. Controls from unsynchronized WT and *sir4Δ* cells transformed with the pRS314-pGAL1-HO plasmid, grown in glycerol-lactate medium lacking tryptophan (-) and exposed to galactose for 1h (G), are displayed on the right. The *HMR* cassette is deleted in the *sir4-AA* and *sir4Δ* cells used here. HO cleavage at *MAT* (in all cells) and *HML* (in *sir4Δ* cells only) creates fragments of chromosome 3, whose positions are indicated on the right of the figure.

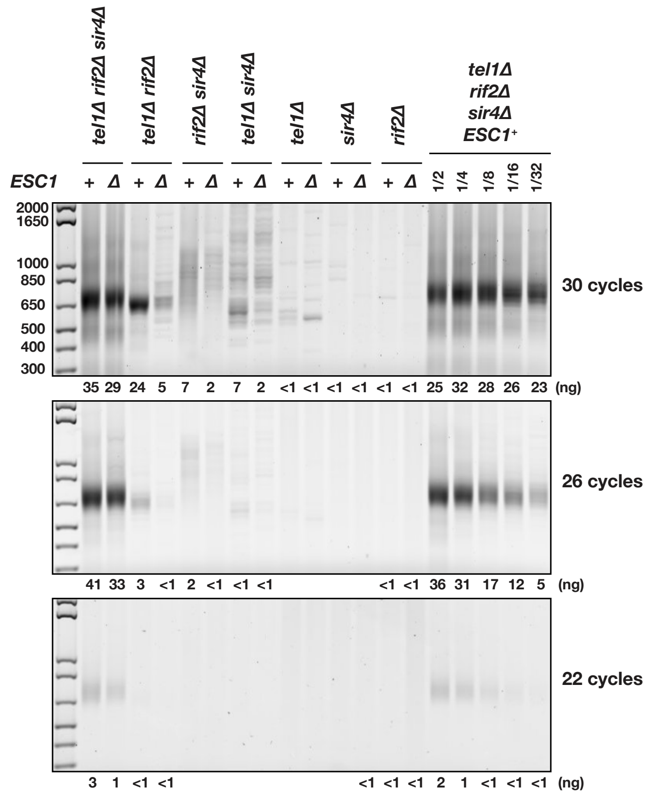

Supplementary Figure S7:

Esc1 is not required for NHEJ inhibition at telomeres in *S. cerevisiae*. Cells were grown exponentially in rich medium and allowed to reach stationary phase in 6 days. Fusions between X and Y' telomeres were amplified by PCR with 30, 26 and 22 cycles. Dilution of the template DNA provides a semi-quantitative estimation of the method's sensitivity. Esc1 loss slightly reduces telomere fusion frequency in contexts where telomere protection is weakened by Tel1, Rif2 or Sir4 losses. This result rules out a role for Esc1 similar to Sir4 in telomere protection in *S. cerevisiae*.

Esc1 *Saccharomyces cerevisiae*

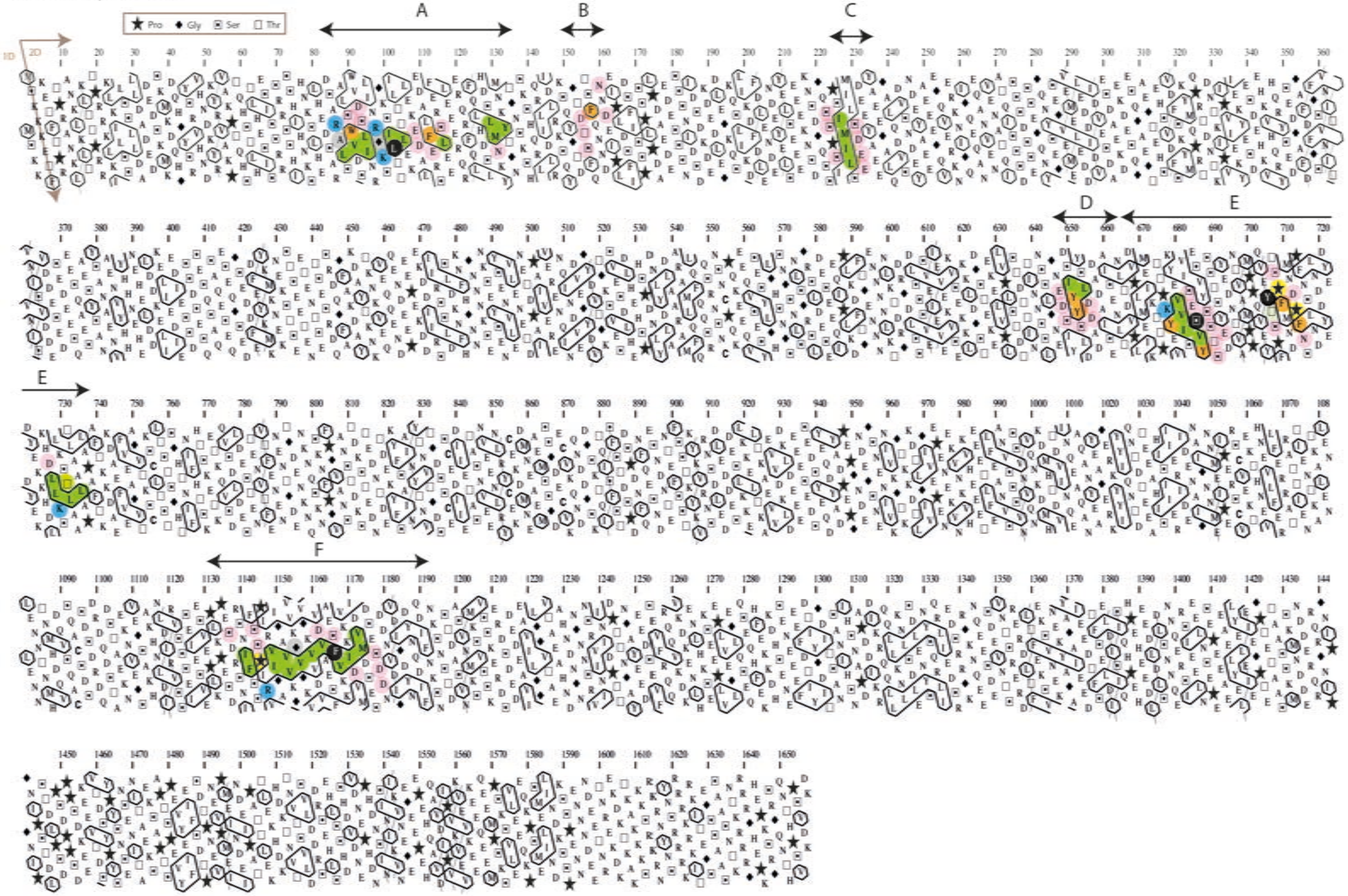

Esc1 *Tetrapisispora blattae*

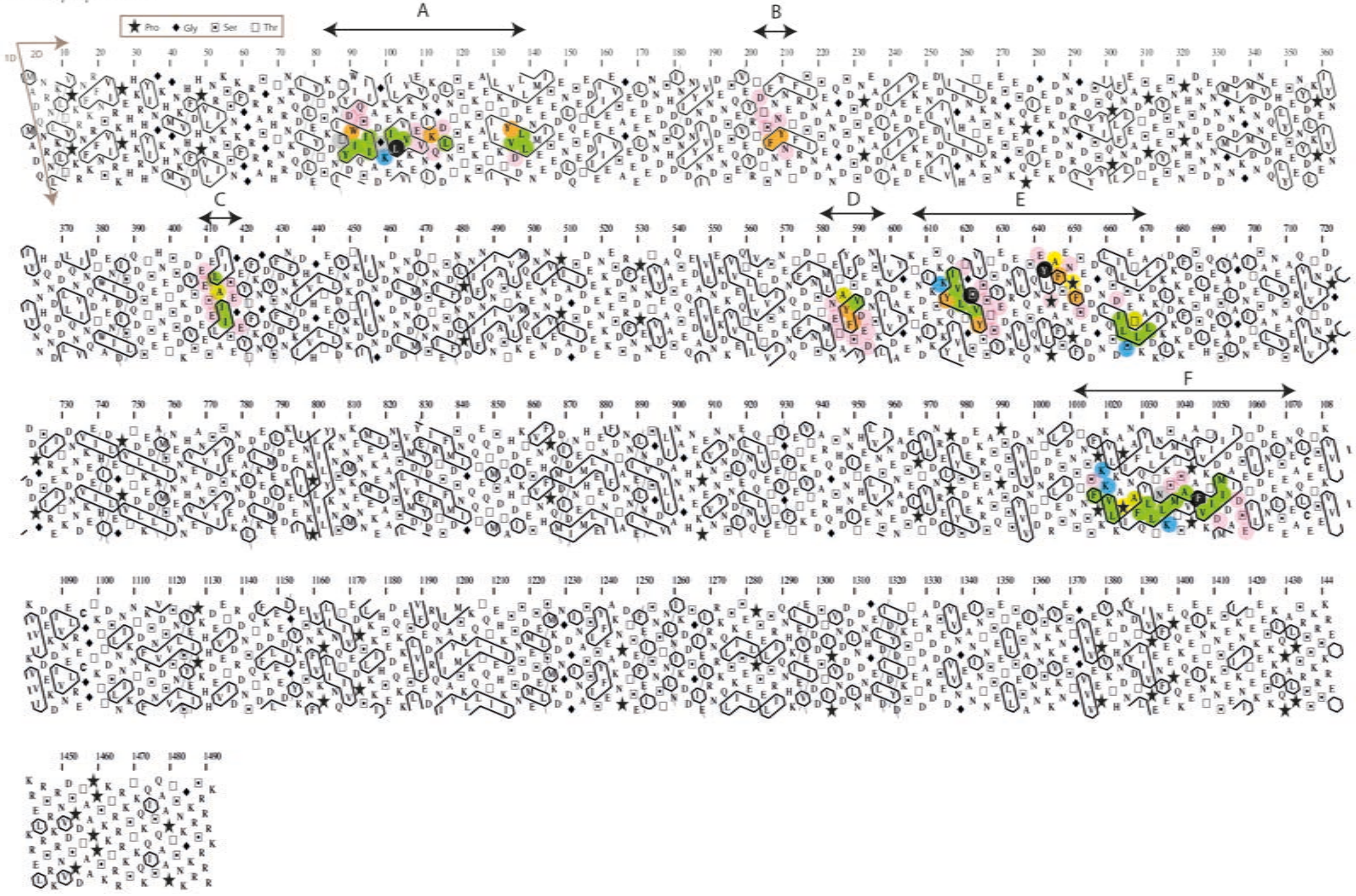

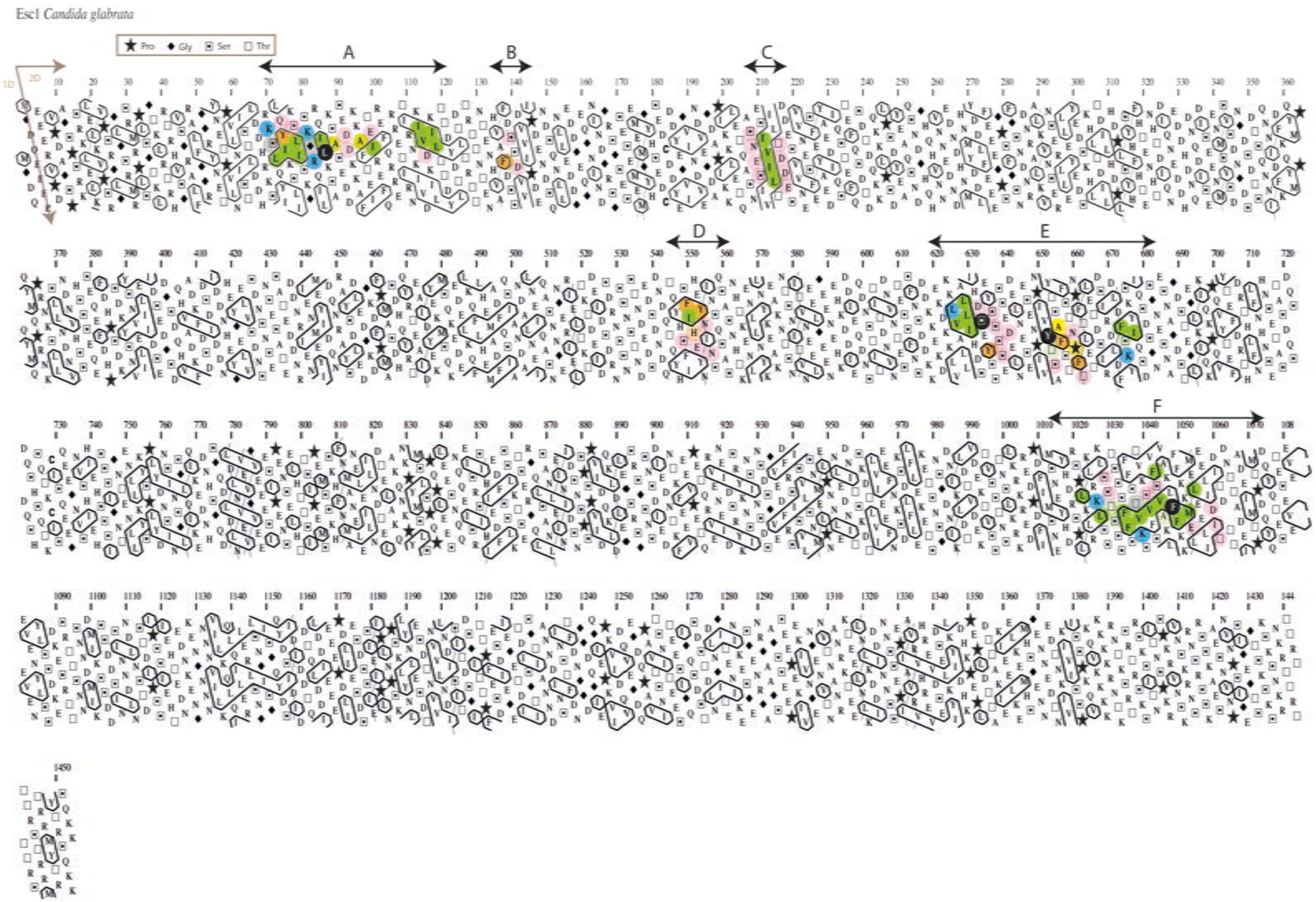

Supplementary Figure S8 (3 pages): HCA plots of the Esc1 proteins from S. *cerevisiae, T. blattae* and *C. glabrata*, illustrating how HCA can help to highlight conserved motifs around conserved hydrophobic clusters, even when they are missed by standard tools due to the high variability of intervening sequences. Here for instance, motifs Esc1-B and -F were found in the *C. glabrata* sequence through examination of its HCA plot. The same is true for motif Esc1-C in the *T. blattae* sequence. No motif Esc1-F could be found in the *Lachancea* and *Eremothecium* sequences. Color code for the conservation of amino acids: green = strong hydrophobic, orange = aromatic, pink = acidic, blue = basic, grey = tiny, yellow = loop-forming (P, G, D, N, S). Arrows indicated the six conserved regions, labelled A to F.

Literature cited

Callebaut I, Labesse G, Durand P, Poupon A, Canard L, Chomilier J, Henrissat B, Mornon JP. 1997. Deciphering protein sequence information through hydrophobic cluster analysis (HCA): current status and perspectives. Cell. Mol. Life Sci. 53:621-645.

Dosztányi Z, Csizmók V, Tompa P, Simon I. 2005a. IUPred: web server for the prediction of intrinsically unstructured regions of proteins based on estimated energy content. Bioinformatics 21:3433-3434.

Dosztányi Z, Csizmók V, Tompa P, Simon I. 2005b. The pairwise energy content estimated from amino acid composition discriminates between folded and intrinsically unstructured proteins. J Mol Biol 347:827-839.

Dosztányi Z, Mészáros B, Simon I. 2009. ANCHOR: web server for predicting protein binding regions in disordered proteins. Bioinformatics 25:2745-2746.

Eudes R, Le Tuan K, Delettré J, Mornon J, Callebaut I. 2007. A generalized analysis of hydrophobic and loop clusters within globular protein sequences. BMC Struct Biol 7:2.

Faure G, Callebaut I. 2013. Comprehensive repertoire of foldable regions within whole genomes. PLoS Comput Biol 9:e1003280.

Gaboriaud C, Bissery V, Benchetrit T, Mornon JP. 1987. Hydrophobic cluster analysis: an efficient new way to compare and analyse amino acid sequences. FEBS Lett. 224:149-155.

Lescasse R, Pobiega S, Callebaut I, Marcand S. 2013. End-joining inhibition at telomeres requires the translocase and polySUMO-dependent ubiquitin ligase Uls1. EMBO J. 32:805-815.

Marcand S, Pardo B, Gratias A, Cahun S, Callebaut I. 2008. Multiple pathways inhibit NHEJ at telomeres. Genes Dev 22:1153-1158.

Mészáros B, Simon I, Dosztányi Z. 2009. Prediction of protein binding regions in disordered proteins. PLoS Comput Biol 5:e1000376.
